# Analysing the Structural Identifiability and Observability of Mechanistic Models of Tumour Growth

**DOI:** 10.3390/bioengineering12101048

**Published:** 2025-09-29

**Authors:** Adriana González Vázquez, Alejandro F. Villaverde

**Affiliations:** 1 Universidade de Vigo, Department of Systems Engineering and Control, 36310 Vigo, Galicia, Spain; adriana.gonzalez@uvigo.gal; 2 Centro de Investigación e Tecnoloxía Matemática de Galicia (CITMAga), 15782 Santiago de Compostela, Galicia, Spain

**Keywords:** mathematical modelling, identifiability, observability, mathematical oncology, tumour growth, radiotherapy, chemotherapy, immunotherapy

## Abstract

Mechanistic cancer models can encapsulate beliefs about the main factors influencing tumour growth. In recent decades, many different types of dynamic models have been used for this purpose. The integration of a model’s differential equations yields a simulation of the behaviour of the system over time, thus enabling tumour progression to be predicted. A requisite for the reliability of these quantitative predictions is that the model is structurally identifiable and observable, i.e., that it is theoretically possible to infer the correct values of its parameters and state variables from time course data. In this paper, we show how to analyse these properties of tumour growth models using a well-established methodology, which we implemented previously in an open-source software tool. To this end, we provide an account of 20 published models described by ordinary differential equations, some of which incorporate the effect of interventions including chemotherapy, radiotherapy, and immunotherapy. For each model, we describe its equations and analyse their structural identifiability and observability, discussing how they are affected by the experimental design. We provide computational implementations of these models, which enable readily reproducing results. Our results inform about the possibility of inferring the parameters and state variables of a given model using a specific measurement setup, and, together with the corresponding methodology and implementation, they can be used as a blueprint for analysing other models not included here. Thus, this paper serves as a guide to select the most appropriate model for each application.

## 1. Introduction

In recent decades, ever-growing progress has been achieved regarding the understanding of cancer biology. The result is an increasingly complex picture, well illustrated by the enlargement of those traits considered to be hallmarks of cancer [1,2,3]. Likewise, the use of mathematical modelling—and, more specifically, dynamic modelling—has become increasingly common in the biomedical sciences. As a result, mathematical oncology has become an established area of mathematical biology, with its own set of hallmarks [4,5]. There are currently a wealth of computational tools that support the biomedical modelling process on the one hand and that enable fully exploiting the capabilities of mathematical models on the other [6]. The integration of mathematical and computational tools—sometimes known as the (dynamic) systems biology approach [7,8,9]—has facilitated the modelling of tumour dynamics and the exploration of the therapeutic efficiency of different treatments.

In this paper, we focus on mathematical models that are *mechanistic*: i.e., they provide explanations of the mechanism by which a phenomenon takes place; and *dynamic*: i.e., they are based on (differential) equations that describe the key aspects of their dynamics. These models can be used for many purposes [10]. First of all, they encapsulate the available knowledge (or beliefs) about a process, enabling results to be produced and to test if a hypothesis is consistent with the current knowledge. Furthermore, they can make quantitative predictions [11], ideally with an estimate of the associated uncertainties. This predictive capability provides many possibilities; crucially, it allows not only to anticipate the result of a treatment but also to optimise its design.

Thus, in the context of mathematical or computational oncology, these models can be used to understand complex phenomena such as the impact of hypoxia on tumour growth, the interactions between tumour and immune cells, and the effects of therapeutic regimens. There are currently a wide variety of tumour growth models of varying mathematical formalisms, purposes, and levels of detail (see, e.g., the reviews [5,12,13,14,15] and the references therein). In this context, Ref. [16] provided a conceptual review of tumour growth dynamics, showing that the growth patterns of the tumours can be grouped into a limited set of categories (exponential, surface, sigmoidal, atypical, and multistep), each reflecting the insights that drive tumour progression. Complementarily, Ref. [17] carried out a systematic comparison of intrinsic growth laws, fitting classical models such as exponential, logistic, Gompertz, von Bertalanffy, and power-law dynamics to experimental data from diverse cancer types. Most previous comparative studies focused either on classifying intrinsic tumour growth laws or fitting them to experimental datasets. Our study differs in scope as we systematically analyse the structural identifiability and observability of mechanistic ODE-based models, including those that integrate therapeutic intervention. One aim of this paper is to provide a compact account of some of the most relevant ones, facilitating appropriate model choice for a given purpose. To this end, we outline a set of mathematical models that describe tumour growth, with and without therapeutic interventions, including radiotherapy, immunotherapy, and chemotherapy. We consider models based on ordinary differential equations (ODEs), which can describe processes that are deterministic and homogeneous. For some of them, stochastic extensions have been published, which enable studying the influence of random fluctuations. We assign an acronym to each model, and we provide their equations in a systematic notation. Table 1 lists the 16 basic models considered in this paper; since we also discuss variants of some of them, the total number of analysed models is 20.

The application of these models goes beyond understanding the biological aspects of cancer; their ultimate goal would arguably be to guide the design and optimisation of treatments, maximising their efficacy while minimising adverse effects for the patient. The use of optimal control in this context has a long history that includes the design of treatments based on chemotherapy [18,19], immunotherapy [20], and radiotherapy [21]. In order to perform successfully in those applications, a model must be properly calibrated [6]. The key tasks of the calibration process include identifiability and observability analysis [22], parameter estimation [23], sensitivity analysis [24], uncertainty quantification [25], experimental design [26], and model selection [27].

In this work, we focus on the first of the tasks in the above list. These analyses assess whether it is theoretically possible to infer the model parameters (*identifiability*) and internal dynamics or state variables (*observability*) from measurable outputs. The relevance of identifiability in clinical applications was recently emphasised by the authors of [28], who illustrated how a lack of identifiability can undermine predictive accuracy even in carefully constructed cancer models. To help avoid these pitfalls, we perform a detailed analysis of the structural identifiability of the parameters of the collected models, and of the observability of their state variables. We provide all the files that enable reproducing the results in the models folder of the STRIKE-GOLDD toolbox, which we used to perform the analyses (https://github.com/afvillaverde/strike-goldd, accessed on 1 August 2025).

The remainder of this paper is organised as follows: Section 2 introduces the methodological framework, including the mathematical formulation of ODE models and the tools used for structural identifiability and observability analysis. Section 3 presents a catalogue of tumour growth models, classified according to the treatments that they include: no therapy, radiotherapy, immunotherapy, or chemotherapy. Section 4 reports and discusses the results of the analyses for each group of models and explains the implications for parameter estimation and model exploitation. Finally, Section 5 summarises the main conclusions and outlines directions for future research.

**Table 1:** Summary of the models considered in this paper.

Model Name	Acronym	Treatment	Reference
Exponential	EXP	None	[5]
Power Law	POW	None	[29]
Lotka–Volterra	L–V	None	[30]
Gompertz	GOM	None	[29]
Logistic	LOG	None	[29]
Von Bertalanffy	BERT	None	[29]
Radio Base	RAD	Radiotherapy	[31]
Carrying Capacity Radio	RCAP	Radiotherapy	[32]
Radio Necrotic	NECR	Radiotherapy	[33]
Immuno CAR-T cells CRS	CRS	Immunotherapy	[34]
Immuno-Hematological CAR-T cells	HCART	Immunotherapy	[35]
Immuno-Radio	IMRAD	Immunotherapy	[36]
CML Tumour-Immune Int	LEUK	Immunotherapy	[37]
Cancer–Immunity Cycle	CYCLE	Chemotherapy	[38]
Cytostatic and Cytotoxic Effects	CYTO	Chemotherapy	[39]
Cancer Immune Chemotherapy Vitamins	CICV	Chemotherapy	[40]

## 2. Methods

In this section, we present the mathematical framework used to describe tumour dynamics based on ordinary differential equations (ODEs). We then introduce the notions of structural identifiability and observability, which are key to determine whether model parameters and internal states can be inferred from measurable outputs. Finally, we describe the computational tools used to perform these analyses, focusing on the STRIKE-GOLDD Matlab toolbox 4.2.2.

### 2.1. Modelling Framework

Ordinary differential equations are the most widely used framework for modelling tumour growth. In their general form, they can be written as

(1) $$M:\left\{\begin{array}{cc} \dot{x}\left(t\right) & =f\left(u(t),x(t),\theta \right),\\ y(t) & =h\left(u(t),x(t),\theta \right), \end{array}\right.$$

where *f* and *h* are analytic functions, which are in general nonlinear; $x\left(t\right)\in R^{n_{x}}$ is the vector of state variables at time *t*; $u\left(t\right)\in R^{n_{u}}$ is the input vector; $y\left(t\right)\in R^{n_{y}}$, the output vector; and $\theta \in R^{n_{\theta }}$ is the parameter vector. In the following, we will sometimes omit the time dependence for ease of notation; i.e., we may simply write x,y,u. A model’s outputs are the measurable functions, which are often a subset of the state variables, or a simple function of them (e.g., a sum). For the models in which we are interested, the inputs are the treatments or therapeutic actions.

### 2.2. Structural Identifiability and Observability

**Figure 1:**
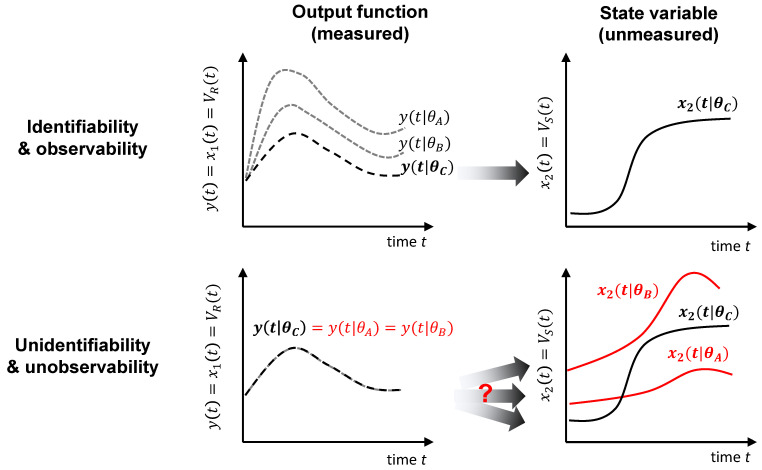
Conceptual representation of structural identifiability and observability. As an example, think of a model describing the time courses of the volumes of two cell populations: one resistant to radiotherapy ($V_{R}\left(t\right)$) and one susceptible to it ($V_{S}\left(t\right)$). We assume that only $V_{R}\left(t\right)$ is measured; i.e., the model output is the corresponding state variable, $y\left(t\right)=x_{1}\left(t\right)=V_{R}\left(t\right),$ whose time course is shown in the first column. The other state variable, $x_{2}\left(t\right)=V_{S}\left(t\right),$ shown in the second column, is unmeasured. The first row illustrates the case in which the model is identifiable and observable: each parameter vector (here represented by three of them, $\theta_{A},\theta_{B},\theta_{C}$) yields a different output. Thus, by measuring $V_{R}\left(t\right)$, we can determine the correct parameter vector (in this example, it is $\theta_{C}$), and, by simulating the model with it, we obtain a single prediction of the unmeasured state variable, $V_{s}\left(t\right),$ which is therefore observable. The second row illustrates the opposite situation: there are an infinite number of parameter vectors that yield the same output, and therefore we cannot tell which one is the correct one (i.e., some or all of the parameters are unidentifiable); since the model simulations with each of these parameter vectors differ in the prediction of the unmeasured state variable $V_{S}\left(t\right),$ we cannot determine which one is correct (i.e., $V_{S}\left(t\right)$ is unobservable).

In this study, we followed a systematic approach to evaluate the structural properties of ODE-based models of the form (1). We focused on two fundamental aspects: structural identifiability and observability, which are essential to ensure the model’s reliability for parameter estimation and model prediction. Structural identifiability refers to the theoretical possibility of uniquely determining the model parameters from ideal noise-free input–output data. When a parameter is not identifiable, there are infinite different parameter sets that result in identical outputs, hindering model calibration and predictive use [22]. A related concept, observability, refers to the ability to infer the internal state variables of the system based on output measurements over time. We refer to structural identifiability and observability jointly with the acronym SIO [41]. Figure 1 illustrates these two concepts.

It should be noted that the SIO properties can be analysed before collecting any data (which is why they are sometimes called a priori properties) since they do not depend on the specific measurement values but only on the output definition. In other words, they are fully determined by Equation (1), and their analysis implicitly assumes that it is possible to measure the output y(t) at any possible instant $t_{i}$ and with perfect precision. Thus, SIO are *necessary* conditions for a model’s successful identification; *sufficient* conditions are provided by their practical counterparts [22,42,43]. *Practical* identifiability analysis requires numerical—not symbolic—methodologies, and it is highly application-dependent, so it is out of the scope of the present study.

Therefore, to assess the way in which the availability of measured outputs influences SIO, in our analyses, we considered in some cases various measurement scenarios, including individual and combined outputs. We explored different measurement configurations, such as isolated versus combined output observations, to understand their impact on the structural properties of the system. For example, if a model describes the evolution over time of the tumour volumes of two different cell populations—one susceptible to radiotherapy and the other one resistant to it—as in the example of Figure 1, the model’s SIO properties may be different if we measure the volume of the susceptible population, of the resistant population, of both of them separately, or of their sum. We would also like to note that in this paper we analyse local versions of structural identifiability and observability. Even though they are local properties, since they are structural they are also generally valid. That is, if a parameter is structurally locally identifiable (SLI), its true value can be distinguished from any other values in a neighbourhood around it, and this result holds *for every possible value* in parameter space. The only possible exceptions to this generic character, if they exist, belong to a set of measure zero; in other words, even if a parameter is SLI, it may become unidentifiable for a particular value, or at most for a finite number of values. Furthermore, empirical studies [44] suggest that the distinction between locally and globally identifiable is typically of little importance in practice since most SLI parameters are also globally identifiable, and for those that are not there is often no possibility of confusion.

### 2.3. Methodology for Analysing Structural Identifiability and Observability

There are several approaches to the analysis of the structural local identifiability and observability (SIO) of a nonlinear model. Here, we use the one based on differential geometry, which was originally proposed in [45], among other works. This approach includes the state variables and parameters in an augmented state vector, $\tilde{x}=\left[x,\theta \right],$ and derives an observability–identifiability matrix $O_{I}^{NL}\left(\tilde{x},u\right)$ of the form [46]

(2) $$\begin{array}{c} O_{I}^{NL}\left(\tilde{x},u\right)=\left(\begin{array}{c} \frac{\partial }{\partial \tilde{x}}h\left(\tilde{x}\right)\\ \frac{\partial }{\partial \tilde{x}}\left(L_{f}h\left(\tilde{x}\right)\right)\\ \frac{\partial }{\partial \tilde{x}}\left(L_{f}^{2}h\left(\tilde{x}\right)\right)\\ \vdots \\ \frac{\partial }{\partial \tilde{x}}\left(L_{f}^{n_{x}+n_{\theta }-1}h\left(\tilde{x}\right)\right) \end{array}\right) \end{array}$$

The above matrix is built from Lie derivatives of the output. The first-order Lie derivative, $L_{f}h\left(\tilde{x},u\right),$ is defined as

(3) $$L_{f}h\left(\tilde{x},u\right)=\frac{\partial h(\tilde{x})}{\partial \tilde{x}}f\left(\tilde{x},u\right)+\sum_{j=0}^{j=\infty }\frac{\partial h(\tilde{x})}{\partial u^{\left(j\right)}}u^{\left(j+1\right)}.$$

Higher-order Lie derivatives are recursively calculated as

(4) $$L_{f}^{i}h\left(\tilde{x}\right)=\frac{\partial L_{f}^{i-1}h\left(\tilde{x}\right)}{\partial \tilde{x}}f\left(\tilde{x},u\right)+\sum_{j=0}^{j=\infty }\frac{\partial L_{f}^{i-1}h\left(\tilde{x}\right)}{\partial u^{\left(j\right)}}u^{\left(j+1\right)}$$

Once the $O_{I}^{NL}\left(\tilde{x}\right)$ matrix has been obtained, we can determine the SIO of a model as follows: if $\mathrm{rank}\left(O_{I}^{NL}\left(\tilde{x}\right)\right)=n_{x}+n_{\theta }$, all the parameters are structurally locally identifiable, and all the state variables are observable. If $\mathrm{rank}\left(O_{I}^{NL}\left(\tilde{x}\right)\right)<n_{x}+n_{\theta }$, the model is not fully identifiable or observable. In this case, we determine the identifiability of each parameter and the observability of each state variable by removing the corresponding matrix column and recalculating the matrix rank, as described, e.g., in [41].

We performed these analyses using the STRIKE-GOLDD toolbox, version 4.2.2 [47], an open-source Matlab toolbox based on differential geometry methods for structural system analysis. This tool enables the examination of model properties independently of specific parameter values or experimental data and is freely available on GitHub. An alternative method is the Matlab toolbox StrucID [48], which is based on the calculation of sensitivities. It is usually faster than STRIKE-GOLDD and can hence be convenient for some analyses. Here, we used it for one model (IMRAD) that was particularly computationally expensive.

Some models (e.g., those that include radiotherapy) involve time-dependent treatment inputs such as impulses, which are often modelled with Dirac delta functions. We treated these impulses as constants during the analysis. This representation enables the study of identifiability and observability with the approach described above without compromising the mathematical consistency of the analysis.

## 3. A Catalogue of Models

In this section, we provide an overview of some representative ODE models of tumour growth found in the literature. We provide their equations, and we classify the variables of each model as inputs (which can be manipulated, and usually correspond to treatments), outputs (i.e., the quantities or functions that can be measured), states (whose variation over time is provided by the model’s differential equations), and parameters (constant quantities, which may be either known or unknown). This classification is a requirement for performing SIO analyses. Table 2 lists the parameters, state variables, inputs, and outputs of the ODE models considered in this paper, along with their acronyms and equations.

### 3.1. Tumour Growth Models Without Therapy

In the following subsection, we explore models that describe tumoural growth in the absence of any type of treatment.

#### 3.1.1. Exponential Model (EXP)

The most basic growth model (EXP1) is the one provided by the following equation [5]:

(5) $$\frac{dV}{dt}=\lambda V$$

Its solution, which is an exponential over time, is

$$V\left(t\right)=V_{0}\exp\left(\lambda t\right),$$

where V(t) is the tumour volume over time, λ the net growth rate of the tumour, and $V_{0}$ the initial volume of the tumour.

While this model can typically accurately describe early growth stages, it fails to describe the reduced growth rates and eventual saturation due to the lack of nutrients and oxygen that vascular tumours develop in vivo, and also in avascular tumours cultured in vitro. To account for these aspects, a slightly more complex model can be used (EXP2):

(6) $$\frac{dV}{dt}=\lambda V\left(1-\frac{V}{K}\right)$$

Its solution is

$$V\left(t\right)=\frac{KV_{0}}{V_{0}+\left(K-V_{0}\right)\exp\left(-\lambda t\right)}$$

with $V\left(0\right)=V_{0}>0$, and K>0 represents the population carrying capacity, which is the value approached by V(t) as time tends to infinity.

The model can be made more flexible by introducing an additional parameter θ, resulting in (EXP3) the following:

(7) $$\frac{dV}{dt}=\frac{\lambda }{\theta }V\left(1-{\left(\frac{V}{K}\right)}^{\theta }\right)$$

which leads to the following solution:

$$V\left(t\right)=K{\left(\frac{V_{0}^{\theta }}{V_{0}^{\theta }+\left(K^{\theta }-V_{0}^{\theta }\right)\exp\left(-\lambda t\right)}\right)}^{\frac{1}{\theta }}$$

The possible output of these models is obviously the tumour volume, which is often measured over time to assess the growth dynamics and response to treatments. A common limitation of these models is the difficulty of relating their parameters to the behaviour of single cells.

#### 3.1.2. Power Law Model (POW)

The Power Law model [29] adopts a different approach for describing how cells actively divide and grow in a lower spatial dimension than the full organoid. It is provided by the equation

(8) $$\frac{dN}{dt}=aN^{\gamma },$$

with 0<γ<1, which has the following explicit solution:

$$N\left(t\right)={\left(N_{0}^{1-\gamma }+\left(1-\gamma \right)at\right)}^{1/(1-\gamma )},\;t\geq 0.$$

where N(t) is the number of cells of an organoid, and *a* is the rate at which the cells divide. This model is useful, for example, when the growth of the tumour is restricted to its surface. Its primary measurable variable—i.e., its possible output—is the population size, which can be interpreted as the tumour volume in certain biological contexts.

#### 3.1.3. Lotka–Volterra Model (L–V)

The classical Lotka–Volterra model [30] can be used to describe the dynamics of several populations, e.g., of radio-sensitive and radio-resistant tumour cells. Without modelling the effect of radiotherapy explicitly, the governing equations of this model can be written as

(9) $$\frac{dV_{S}}{dt}=\lambda_{S}V_{S}\left(1-\frac{V_{S}}{K_{S}}-\gamma_{R}\frac{V_{R}}{K_{S}}\right)$$

(10) $$\frac{dV_{R}}{dt}=\lambda_{R}V_{R}\left(1-\frac{V_{R}}{K_{R}}-\gamma_{S}\frac{V_{S}}{K_{R}}\right)$$

where $V_{S}\left(t\right)$ and $V_{R}\left(t\right)$ represent the volumes of the control and resistant populations, $\lambda_{S}$ and $\lambda_{R}$ their growth rates, and $K_{S}$ and $K_{R}$ their carrying capacities. Additionally, $\gamma_{S}$ and $\gamma_{R}$ describe the effect that radiosensitive cells have on resistant cells and vice versa.

The possible outputs for this model are the tumour volumes of the two interacting populations, $V_{S}$ and $V_{R}$. These volumes represent the measurable variables, capturing the growth dynamics of each tumour population over time. It may be possible to measure each of these volumes individually, although it is easier to obtain measurements of their sum, $V_{S}+V_{R}$. To this end, one promising approach is the use of dual-reporter systems in preclinical models for in vivo systems. Here, the sensitive and resistant clones are genetically labelled with distinct fluorescent proteins, enabling the tracking of each subpopulation through intravital microscopy or fluorescence molecular tomography [49].

#### 3.1.4. Gompertz Model (GOM)

This well-known model [29] assumes that an initial growth rate a>0 decreases exponentially within time, according to a decay parameter b≥0. The differential equation is (GOM1) as follows:

(11) $$\frac{dN}{dt}=ae^{-bt}N,$$

which, for $N\left(0\right)=N_{0}$, has the solution

$$N\left(t\right)=N_{0}\exp\left(\frac{a}{b}\left(1-\exp\left(-bt\right)\right)\right),\;t\geq 0$$

There is an alternative formulation of this model (GOM2) that considers an initial growth rate α and a carrying capacity *K* as follows:

(12) $$\frac{dN}{dt}=\alpha \log\left(\frac{K}{N}\right)N=\alpha \log\left(K\right)N-\alpha N\log\left(N\right)$$

Its solution is

$$N\left(t\right)=K\exp\left(\log\left(\frac{N_{0}}{K}\right)\exp\left(-\alpha t\right)\right),\;t\geq 0$$

The possible output for both versions of the Gompertz model is the population size, *N*.

#### 3.1.5. Logistic Model (LOG)

A model similar to the previous one is the so-called logistic model, where the growth rate decays linearly with the size of the population until the population reaches the carrying capacity *K*; it is provided by the equation

(13) $$\frac{dN}{dt}=aN\left(1-\frac{N}{K}\right)$$

where $N\left(0\right)=N_{0}$, and with solution

$$N\left(t\right)=\frac{N_{0}K}{N_{0}+\left(K-N_{0}\right)\exp\left(-at\right)},\;t\geq 0$$

The main output is obviously the population size, *N*, which can be measured over time as the population grows and approaches its limit, *K*.

#### 3.1.6. Von Bertalanffy Model (BERT)

The model proposed by von Bertalanffy [29] assumes growth as a three-dimensional ball; cell division happens only on the surface, and the deaths are uniformly distributed across the organoid at a rate b>0. This model is provided by the equation

$$\frac{dN}{dt}=aN^{2/3}-bN,$$

with $N\left(0\right)=N_{0}$. It has another more general version that replaces the exponent 2/3 with a parameter γ. Thus, it considers that the subset of actively dividing cells occupies a lower spatial dimension. This variant has the equation

(14) $$\frac{dN}{dt}=aN^{\gamma }-bN$$

which, for 0<γ<1, has the explicit solution

$$N\left(t\right)={\left(\left(\frac{a}{b}\right)+\left(N_{0}^{1-\gamma }-\frac{a}{b}\right)\exp\left(-(1-\gamma )bt\right)\right)}^{\frac{1}{1-\gamma }},\;t\geq 0$$

reaching the carrying capacity $K:={\left(\frac{a}{b}\right)}^{\frac{1}{1-\gamma }}$ as t→∞. Reparameterising in terms of *b*, γ, and *K*, it can be rewritten as

$$N\left(t\right)=K{\left(1+\left({\left(\frac{N_{0}}{K}\right)}^{1-\gamma }-1\right)\exp\left(-(1-\gamma )bt\right)\right)}^{\frac{1}{1-\gamma }},\;t\geq 0$$

### 3.2. Tumour Growth Models with Radiotherapy

In this subsection, we list models that describe the way in which radiotherapy affects tumour growth.

#### 3.2.1. Radio Base Model (RAD)

This model was presented in [31] and is provided by (RAD1)

(15) $$\frac{dV}{dt}=\lambda V-\sum_{i=1}^{n}\left(\alpha d+\beta d^{2}\right)V\delta \left(t-t_{i}\right)$$

with solution for $t_{i}^{+}\leq t\leq t_{i+1}^{-}$:

$$V\left(t\right)=V\left(t_{i}^{+}\right)\exp\left[\lambda (t-t_{i})\right]$$

where $V\left(t_{i}^{+}\right)=V\left(t_{i}^{-}\right)\exp\left[-n(\alpha d+\beta d^{2})\right]$, *n* is the number of radiotherapy fractions, *d* the dose administered at times $t_{i}$ (i=1,2,3,…,n), and δ(t) denotes the Dirac delta function, defined such that, for an arbitrary smooth function f(t),

$$\int_{-\infty }^{+\infty }f\left(t\right)\delta \left(t-t_{i}\right)dt=f\left(t_{i}\right).$$

$V(t_{i}^{-})$ and $V(t_{i}^{+})$ represent the tumour volumes immediately before and after radiotherapy, and the survival fraction (SF) is defined as $SF=V\left(t_{i}^{+}\right)/V\left(t_{i}^{-}\right)$, so

$$SF=\frac{V(t_{n}^{+})}{V(0)}=\exp\left(\lambda t_{n}\right)\cdot \exp\left[-n(\alpha d+\beta d^{2})\right].$$

In order to analyse the model with the approach described in Section 2, the input must be infinitely differentiable. Hence, in our analyses, we replace the delta functions with a constant input that quantifies the average effect of a radiotherapy dose over a short interval. Note that, while this is an approximation, it is arguably closer to reality than a Dirac delta function itself, which cannot be physically realised in practice.

Cell death resulting from radiotherapy can be integrated with the logistic and generalised logistic growth models in the following way (RAD2):

(16) $$\frac{dV}{dt}=\lambda V\left[1-{\left(\frac{V}{K}\right)}^{\theta }\right]-\sum_{i=1}^{n}\left(\alpha d+\beta d^{2}\right)V\delta \left(t-t_{i}\right)$$

with $V\left(0\right)=V_{0}>0$, and θ=1 for the logistic growth law. The possible output for both models is the tumour volume *V*, which can be measured over time as the tumour grows and responds to the doses of radiation.

#### 3.2.2. Carrying Capacity Radio Model (RCAP)

Other authors [32] have proposed a model in which the doses of radiotherapy instantaneously reduce the carrying capacity of the tumour in the logistic model instead of affecting the tumour volume:

(17) $$\frac{dV}{dt}=\lambda V\left(1-\frac{V}{K}\right)-\sum_{i=1}^{n}\gamma V\left(1-\frac{V}{K}\right)\delta \left(t-t_{i}\right)$$

where γ describes how effective a given radiation dose is at killing the proliferative portion of the tumour. It quantifies the effect of radiotherapy on the tumour’s carrying capacity, rather than on its volume, because radiation damage affects both tumour vasculature and its microenvironment. In this way, after each dose, the maximum tumour size is effectively being reduced. The possible output for this model is the tumour volume *V*, which can be measured over time as the tumour grows and responds to radiotherapy.

#### 3.2.3. Radio Necrotic Model (NECR)

This ODE model [33] distinguishes between viable tumour volume, $V_{t}\left(t\right)$, and dead or necrotic tissue, $N_{t}\left(t\right)$, and it involves the equations

(18) $$\frac{dV_{t}}{dt}=\lambda V_{t}\left(1-\frac{V_{t}}{K}\right)-\eta V_{t}-\gamma \sum_{i=1}^{n}V_{t}\delta \left(t-t_{i}\right)$$

(19) $$\frac{dN_{t}}{dt}=\eta V_{t}-\zeta N_{t}+\gamma \sum_{i=1}^{n}V_{t}\delta \left(t-t_{i}\right)$$

where η is the constant rate under which cells undergo necrosis, and ζ is the rate at which the necrotic material undergoes exponential decay. In addition, γ is the death rate of tumour volume *V*. The possible outputs for this model are the viable tumour volume $V_{t}$ and the necrotic material $N_{t}$.

### 3.3. Tumour Growth Models with Immunotherapy

Here, we describe models of immunotherapy, a family of treatments based on the idea of administering drugs to help the immune system fight cancer.

#### 3.3.1. Immuno CAR-T Cells Cytokine Release Syndrome Model (CRS)

In [34], a multi-layer mathematical model that describes the dynamics of CAR-T cell therapy and the associated cytokine release syndrome (CRS) was presented.

The first variable represents the CAR-T cells that were administered to the patient (injected CAR-T cells, $C_{I}\left(t\right)$) to combat cancer. Their dynamics is provided by

(20) $$\frac{dC_{I}}{dt}=-\eta F\left(T_{P}\right)C_{I}-\mu_{I}C_{I}$$

where $F(T_{P})$ represents the antigen–receptor binding interaction that is described by

$$F\left(T_{P}\right)=\frac{T_{P}}{A+T_{P}}$$

The second compartment includes the CAR-T cells that have been activated and are in the process of proliferating (expanding CAR-T cells, $C_{E}\left(t\right)$). These cells multiply in response to the presence of tumour cells that express the specific antigen that they recognise. Their equation is

(21) $$\frac{dC_{E}}{dt}=\nu F\left(T_{P}\right)C_{I}+\kappa F\left(T_{P}\right)C_{E}-\epsilon \left(1-F\left(T_{P}\right)\right)C_{E}+\theta F\left(T_{P}\right)C_{P}-\mu_{E}C_{E}$$

The third compartment represents CAR-T cells that survived the expansion phase and differentiated into memory cells (persistent CAR-T cells, $C_{P}\left(t\right)$). They are important for the long-term immune response as they can be reactivated if needed. Their equation is as follows:

(22) $$\frac{dC_{P}}{dt}=\epsilon \left(1-F\left(T_{P}\right)\right)C_{E}-\theta F\left(T_{P}\right)C_{P}-\mu_{P}C_{P}$$

The fourth compartment represents the tumour cells that express the specific antigen that the CAR-T cells are designed to recognise and attack (antigen-positive tumour cells, $T_{P}\left(t\right)$); this variable describes how CAR-T cells affect the tumour burden:

(23) $$\frac{dT_{P}}{dt}=\rho T_{P}\left(1-\frac{T_{P}+T_{N}}{K}\right)-\gamma \frac{C_{E}}{B+C_{E}}T_{P}$$

Likewise, the dynamics of antigen-negative tumour cells ($T_{N}\left(t\right)$) is provided by

(24) $$\frac{dT_{N}}{dt}=\rho T_{N}\left(1-\frac{T_{P}+T_{N}}{K}\right)g_{0}\gamma \frac{C_{E}}{B+C_{E}}T_{N}$$

In this case, $g_{0}$ is a constant fraction that represents a reduced cytotoxicity due to antigen absence.

The model parameters are *A*, the half-saturation constant; $\mu_{I}$, $\mu_{E}$, and $\mu_{P}$, the death rates of injected, expanding, and persistent CAR-T cells, respectively; ρ, the tumour growth rate; and *K*, the tumour cell carrying capacity.

An additional layer describes cytokine dynamics and cytokine release syndrome (CRS). It assumes that cytokine release is mediated by macrophage activation, driven by three main mechanisms: the release of inflammatory mediators by activated CAR-T cells, the release of DAMPs (Damage-Associated Molecular Patterns, which are molecules released by stressed or dying cells that trigger immune activation) from tumour cell death, and contact-dependent activation between macrophages and CAR-T cells via CD40/CD40–ligand interaction. Under these assumptions, the model for macrophage activation and cytokine release reads as

(25) $$\frac{dM_{i}}{dt}=\sigma_{M}-h\left(C_{E},T_{P},M_{a}\right)M_{i}-\delta_{M}M_{i}$$

(26) $$\frac{dM_{a}}{dt}=h\left(C_{E},T_{P},M_{a}\right)M_{i}-\delta_{M}M_{a}$$

(27) $$\frac{d{\mathrm{IL}}_{6}}{dt}=\sigma_{I}+\alpha M_{a}-\delta_{I}{\mathrm{IL}}_{6}$$

where $M_{i}$ and $M_{a}$ are the number of naive (monocytes) and activated macrophages at time *t*, ${\mathrm{IL}}_{6}$ is the ${\mathrm{IL}}_{6}$ concentration, $\sigma_{M}$ is the natural production rate of naive macrophages, $\delta_{M}$ is the death rate of macrophages, $\sigma_{I}$ the endogenous production rate of ${\mathrm{IL}}_{6}$, $\delta_{I}$ the ${\mathrm{IL}}_{6}$ natural decay rate, and α the rate of ${\mathrm{IL}}_{6}$ release by activated macrophages, whose activation rate is provided by

$$h\left(C_{E},T_{P},T_{N},M_{a}\right)=\beta_{B}\frac{T_{P}}{A+T_{P}}C_{E}+\beta_{K}\frac{C_{E}}{B+C_{E}}\left(T_{P}+g_{0}T_{N}\right)+\beta_{C}\frac{M_{a}}{C+M_{a}}C_{E}$$

where $\beta_{B}$ is the contribution of the antigen-binding-release of inflammatory signals by CAR-T cells, $\beta_{K}$ is the contribution of the tumour-killing-mediated release of DAMPs, and $\beta_{C}$ the contribution of CAR-T cell–macrophage contact.

Some of the eight state variables in the model are easier to measure than others. We have considered the set $C_{E}$, $M_{a}$, $M_{i}$, and ${\mathrm{IL}}_{6}$ as reasonable outputs; we justify this decision as follows.

Regarding the expanding CAR-T cells ($C_{E}$), the measurement of the expansion of these cells is essential to check the immediate effectiveness of the treatment and to determine how well the immune system is adapting to fight the tumour; this can be conducted through blood tests using flow cytometry, a relatively common procedure in immunotherapy. However, this process still requires specialised equipment and technical personnel.

One important variable in the model is the concentration of the cytokine ${\mathrm{IL}}_{6}$, which plays a major role in the immune and inflammatory responses. ${\mathrm{IL}}_{6}$ is commonly measured to track immune activity in the context of CAR-T therapy, where excessive immune activation can lead to CRS [34]. ${\mathrm{IL}}_{6}$ levels are relatively easy to measure through blood tests (ELISA). This makes ${\mathrm{IL}}_{6}$ one of the more straightforward and low-effort variables to monitor while providing critical insight into the immune response and potential toxicities associated with treatment.

Another output could be the activated macrophages ($M_{a}$) and monocytes ($M_{i}$), whose activity helps to assess the broader immune response beyond the CAR-T cells themselves. These types of cells can be measured through blood tests and flow cytometry, but identifying and distinguishing between them requires additional markers, so the process would be more complex.

Lastly, tumour cells with antigen-positive ($T_{P}$) and antigen-negative ($T_{N}$) expression can indicate whether resistance to the treatment is developing. Unfortunately, measuring these variables often involves more invasive techniques, such as biopsies or advanced imaging, to detect changes in tumour antigen expression. Hence, even though it could be interesting to know these levels, it may require too much effort.

#### 3.3.2. Immuno-Hematological CAR-T Cell Model (HCART)

This model [35] describes the interaction between tumour cells and CAR-T cells (effector and memory populations) in immunodeficient mouse models. The model incorporates tumour growth, CAR-T cell dynamics, and the formation of immunological memory. It uses three ODEs to explore the factors influencing therapy outcomes, including cytotoxicity, tumour-induced immunosuppression, and long-term memory formation.

Effector CAR-T cells ($C_{T}$) proliferate at a certain rate (ϕ) in response to tumour burden, die or differentiate into memory cells (ρ), are activated by interaction with tumour cells (θ), and are suppressed by tumour-induced immunosuppressive mechanisms (α):

(28) $$\frac{dC_{T}}{dt}=\phi C_{T}-\rho C_{T}+\theta TC_{M}-\alpha TC_{T}$$

Memory CAR-T cells ($C_{M}$) are generated from effector CAR-T cells (ϵ), activated back into effector cells when interacting with tumour cells (θ), and decay naturally at a rate μ:

(29) $$\frac{dC_{M}}{dt}=\epsilon C_{T}-\theta TC_{M}-\mu C_{M}$$

Tumour cells (*T*) grow logistically with a maximum growth rate *r* and carrying capacity $\frac{1}{b}$. They are eliminated by effector CAR-T cells through a cytotoxic effect (γ):

(30) $$\frac{dT}{dt}=rT\left(1-bT\right)-\gamma C_{T}T$$

#### 3.3.3. Immuno-Radio Model (IMRAD)

This model [36] describes the tumour response to radio-immunotherapy, i.e., the combination of radiotherapy (RT) with monoclonal antibodies such as αPDL1 and αCTLA4. This model describes the dynamics of tumour cell populations, T-cells, and antibody release.

The equations of the dynamics of tumour cells (*C*), damaged tumour cells ($C_{d}$), T-cells ($T_{a}$), and antibody release (*A*) are, respectively,

(31) $$\frac{dC}{dt}\left(t\right)=\lambda_{1}C\left(t\right)\left(1-\lambda_{2}C_{tot}\left(t\right)\right)-K_{C}\left(t\right)-p\left(1+p_{1}\left(t\right)\right)\frac{{\left(T_{a}\left(t\right)/C_{tot}\left(t\right)\right)}^{q}}{s+{\left(T_{a}\left(t\right)/C_{tot}\left(t\right)\right)}^{q}}C\left(t\right)$$

(32) $$\frac{dC_{d,i}}{dt}\left(t\right)=K_{C}\left(t\right)-\phi \omega \left(\bar{t_{i}}\right)\left(t\right)-p\left(1+p_{1}\left(t\right)\right)\frac{{\left(T_{a}\left(t\right)/C_{tot}\left(t\right)\right)}^{q}}{s+{\left(T_{a}\left(t\right)/C_{tot}\left(t\right)\right)}^{q}}C_{d,i}\left(t\right)$$

(33) $$\frac{dT_{a}}{dt}\left(t\right)=-K_{T}\left(t\right)+a\hat{A}\left(t-\tau_{2}\right)-\hat{T}\left(t-\tau_{2}\right)-\iota T_{a}\left(t\right)C_{tot}\left(t\right)-\eta T_{a}\left(t\right)$$

(34) $$\frac{d\hat{A}}{dt}\left(t\right)=\rho C_{tot}\left(t-\tau_{1}\right)+\psi \phi \sum_{i}\omega \left(\hat{t_{i}}-\tau_{1}\right)C_{d,i}\left(t\right)-\tau_{1}-\sigma \hat{A}\left(t\right)-a\hat{A}\left(t\right)\hat{T}\left(t\right)-\frac{b}{1+c_{4}\left(t\right)}\hat{A}\left(t\right)\hat{T}\left(t\right)$$

In addition, these three equations are needed to fully define the model:

(35) $$\frac{dc_{4}\left(t\right)}{dt}=i_{c_{4}}\left(t\right)\delta \left(t-\left\{t_{c_{4}}\right\}\right)-\nu c_{4}\left(t\right)$$

(36) $$\frac{dp_{1}\left(t\right)}{dt}=i_{p_{1}}\left(t\right)\delta \left(t-\left\{t_{p_{1}}\right\}\right)-\mu p_{1}\left(t\right)$$

(37) $$\frac{d\hat{T}\left(t\right)}{dt}=-a\hat{A}\left(t\right)\hat{T}\left(t\right)-\frac{b}{1+c_{4}\left(t\right)}\hat{A}\left(t\right)\hat{T}\left(t\right)+h$$

#### 3.3.4. CML Tumour-Immune Interaction Model (LEUK)

This model [37] analyses the interactions between Chronic Myeloid Leukemia (CML) cells and immune effector cells, particularly in predicting outcomes after tyrosine kinase inhibitor (TKI) treatment cessation. The model integrates CML cell dynamics with immune responses, incorporating parameters for immune cell functionality and tumour load.

The main equations describing the model are as follows.

Growth of active tumour cells, *T*:

(38) $$\frac{dT}{dt}=p_{T}T\left(1-\frac{T}{k_{T}}\right)-p_{NA}T+p_{AN}T_{N}-e\left(t\right)T-f\left(T\right)h\left(T\right)E$$

Quiescent tumour cells, $T_{N}$:

(39) $$\frac{dT_{N}}{dt}=-p_{AN}T_{N}+p_{NA}T$$

Immune effector cells, *E*:

(40) $$\frac{dE}{dt}=p_{E}-d_{E}E-g\left(T\right)E$$

Functional tumour-immune interaction as a function of tumour load:

(41) r(T)=f(T)E(T)h(T)

Anti-tumour activity of immune cells:

(42) $$f\left(T\right)=\frac{sT}{1+t_{HR}sT}=\frac{m_{K}T}{C_{K}+T^{\prime }}$$

Suppression of immune function by tumour burden:

(43) $$h\left(T\right)=\frac{c_{F}^{2}+f_{MIN}T^{2}}{c_{F}^{2}+T^{2}}$$

Tumour-dependent modulation of immune recruitment:

(44) $$g\left(T\right)=\frac{g_{MAX}T^{2}}{c_{R}^{2}+T^{2}}$$

Immune effector cells as a function of tumour load:

(45) $$E_{S}\left(T\right)=\frac{p_{E}\left(c_{R}^{2}+T^{2}\right)}{d_{E}\left(c_{R}^{2}+T^{2}\right)+g_{MAX}T^{2}}$$

The model parameters are $p_{T}$, the net proliferation rate of active tumour cells; $k_{T}$, the carrying capacity of tumour cells; $p_{NA}$, the rate at which active tumour cells enter quiescence; $p_{AN}$, the rate at which quiescent tumour cells re-enter the active state; e(t), the TKI-induced cell kill rate (dependent on treatment timing); f(T), the immune cell activity per tumour cell; h(T), the suppression factor of immune function by tumour load; $p_{E}$, the production rate of immune effector cells; $d_{E}$, the death rate of immune effector cells; and g(T), the tumour-mediated suppression of immune cell recruitment.

### 3.4. Tumour Growth Models with Chemotherapy

#### 3.4.1. Cancer–Immunity Cycle Model (CYCLE)

A recent model [38] considers the synchronisation of chemotherapy and immunotherapy to enhance treatment efficacy within the cancer–immunity cycle. It adopts an evolutionary game theory approach where healthy cells, cancer cells, and T-cells compete in a non-transitive dynamic, similar to a rock–paper–scissors game, modulated by the tumour microenvironment (*TME*). The aim is to explore how synchronising dosing intervals with the fundamental period of the cancer–immunity cycle ($P_{CIC}$) can improve treatment effectiveness, allowing for lower doses with reduced toxic effects. The main equations describing the model are as follows:

Replicator equations for healthy cell ($x_{1}$) and cancer cell ($x_{2}$) fractions:

(46) $${\dot{x}}_{1}=\left(f_{1}-\langle f\rangle \right)x_{1}$$

(47) $${\dot{x}}_{2}=\left(f_{2}-\langle f\rangle \right)x_{2}$$

Growth of T-cells (*n*):

(48) $$\dot{n}=\varepsilon n\left(1-n\right)h\left(x_{2};\theta \left(t\right)\right)$$

where

(49) $$h\left(x_{2};\theta \left(t\right)\right)=\theta \left(t\right)x_{2}+\left(x_{2}-1\right)$$

Fitness function for each subpopulation (healthy cells and cancer cells):

(50) $$f_{1}={\left(A\left(n\right)\cdot x\right)}_{1}$$

(51) $$f_{2}=C\left(t\right)+\left(1-C\left(t\right)\right){\left(A\left(n\right)\cdot x\right)}_{2}$$

(52) $$\langle f\rangle =x_{1}f_{1}+x_{2}f_{2}$$

Tumour volume growth (V(t)):

(53) $$\dot{V}\left(t\right)=\left(\delta +\alpha_{G}\right)V\left(1-\frac{V}{K}\right)$$

Instantaneous growth rate ($\alpha_{G}$):

(54) $$\alpha_{G}=f_{2}-\langle f\rangle$$

Interpolated payoff matrix:

(55) $$A\left(n\right)=\left(1-g\left(n;a\right)\right)A_{G}+g\left(n;a\right)A_{R}$$

where

(56) $$g\left(n;a\right)=\tanh\left(\frac{n}{a}\right)$$

The variables in these equations are as follows: $x_{1}$ and $x_{2}$ are the fractions of healthy and cancer cells in the total population, respectively. The variable *n* represents the T-cell response level, which indicates immune activity. The chemotherapy control variable, C(t), ranges from 0 (no chemotherapy) to 1 (maximum chemotherapy), while the immunotherapy control variable θ(t) takes values between 2 (no immunotherapy) and higher values to indicate active immunotherapy. The timescale parameter ε determines the relative speed of the immune response compared to cancer cell dynamics, and δ represents the natural immunity’s limitation in counteracting tumour growth entirely. The parameter $\alpha_{G}$ is the instantaneous growth rate of the cancer cell fraction, and *K* represents the carrying capacity or upper limit of tumour volume. The matrices $A_{G}$ and $A_{R}$ are the payoff matrices for tumour growth and regression, respectively, while g(n;a) is an interpolation function that modulates the transition between tumour growth and regression based on immune response level.

#### 3.4.2. Tumour Evolution with Cytostatic and Cytotoxic Effects (CYTO)

This recent model presented by [39] includes the dynamics of tumour volume V(t), which are governed by two key parameters: *a* and *b*.

The parameter a>0 represents the cytostatic effect; it inhibits tumour growth without necessarily causing its reduction, so it acts like a mechanism of slowing down the proliferation of the tumour cells.

The parameter b≥0, on the other hand, corresponds to the cytotoxic effect, modelling the active destruction of tumour cells. When b>0, the model accounts for tumour regression, typically due to the action of chemotherapy or the effect of the immune system. The equation of the model is

(57) $$\frac{d^{2}V}{dt^{2}}-\frac{2}{V}{\left(\frac{dV}{dt}\right)}^{2}+\left[\dot{\phi }\left(a-b\right)-\frac{\ddot{\phi }}{\dot{\phi }}\right]\frac{dV}{dt}+{\left(\dot{\phi }\right)}^{2}abV=0$$

Both parameters (*a* and *b*) interact with a time-scaling function ϕ(t), whose derivatives $\dot{\phi }$ and $\ddot{\phi }$ control how fast the tumour grows or shrinks over time. Since ϕ is assumed to be a known and smooth function, for the purpose of structural identifiability analysis, we treated it as a known input. The interplay between *a*, *b*, and the shape of ϕ(t) determines whether the tumour volume V(t) grows, stabilises, or decreases over time.

To assess the SIO of this model, it is necessary to rewrite it as a system of first-order derivatives, resulting in the following two equations (CYTO2):

(58) $$\frac{dV}{dt}=x_{2}$$

(59) $$\frac{dx_{2}}{dt}=\frac{2}{V}x_{2}^{2}+\left(\dot{\phi }\left(a-b\right)-\frac{\ddot{\phi }}{\dot{\phi }}\right)x_{2}-{\left(\dot{\phi }\right)}^{2}abV$$

#### 3.4.3. Cancer Growth Model with Chemotherapy and Boosting of the Immune System (CICV)

The CICV model presented by [40] describes the interaction between tumour cells C(t) and immune cells I(t), incorporating the effects of chemotherapy and vitamin intake. It is defined as the following system of ODEs:

(60) $$\frac{dC}{dt}=r_{1}C\left(1-r_{2}C\right)-\frac{\alpha_{1}CI}{k_{1}+C}-\beta_{1}C$$

(61) $$\frac{dI}{dt}=\delta -\alpha_{2}CI+\frac{\alpha_{3}C^{2}I}{C^{2}+k_{2}}-\beta_{2}I+\gamma$$

The parameter $r_{1}$ is the tumour’s growth rate, $r_{2}$ defines the inverse of its carrying capacity, $\alpha_{1}$ is the immune killing rate, and $k_{1}$ is a Michaelis–Menten-type saturation constant. Chemotherapy is represented by $\beta_{1}$, the rate at which it kills tumour cells directly. The parameter δ represents the baseline production rate of immune cells, $\alpha_{2}$ quantifies the suppression that tumour cells conduct on the immune system, and $\alpha_{3}$ models the stimulation of immune cells due to tumour presence, following a Holling type III response, which is modulated by the constant $k_{2}$. The parameter $\beta_{2}$ is the death of immune cells due to natural and treatment causes. Finally, γ represents the rate at which regular vitamin intake enhances immune cell production.

## 4. Results and Discussion

In this section, we report the results of the analyses of structural identifiability and observability of the models described in the previous section. The results are summarised in Table 3, Table 4, Table 5 and Table 6. Each model is analysed at least once, and possibly more (for multiple output configurations, indicated in the columns ‘Measured Outputs’) if there are several feasible options.

Table 3 shows the results for models without treatment. All of them are fully identifiable and observable, although it is important to note that, in the case of the Lotka–Volterra model, it is necessary to measure the sum of both volumes (or, naturally, both of them separately, if possible) to achieve identifiability.

Table 4 shows the results for the radiotherapy models. Here, the most remarkable outcome is the realisation that neither the RAD1 nor RAD2 variants are identifiable. The remaining models do not exhibit identifiability issues.

Table 5 shows the results of the immunotherapy models. These models are typically complex, incorporating multiple immune components and a larger number of parameters. This feature is prone to induce unidentifiability issues due to overparameterisation. In the CRS model, only 13 out of the 23 parameters are identifiable, and none of the state variables are observable. HCART has slightly better structural properties, with *T* being observable and measured, but with $C_{T}$ and $C_{M}$ unobservable and several parameters remaining unidentifiable. IMRAD is unobservable, with several parameters (*p*, ϕ, *b*, τ1, τ2, $\omega_{i}$, $\delta_{c4}$, and $\delta_{p1}$) and the initial conditions c4(0),p1(0) being non-identifiable from outputs *C* and *A*. To improve identifiability, we would need to measure more system outputs or fix some parameter values. In the LEUK model, only three out of ten parameters ($p_{NA}$, $p_{AN}$, and $p_{E}$) are identifiable, even if all its states (*T*, $T_{N}$, and *E*) are directly measured. It should be noted that, for liquid cancers such as leukemia, the underlying biology often involves nonlocal interactions between spatially separated compartments, for instance between bone marrow niches and circulating blood cells. In these cases, nonlocal or delay-based models have been proposed to capture the distributed nature of hematopoietic regulation [50,51].

Lastly, Table 6 summarises the results of the chemotherapy models. In the CYCLE model, key system parameters such as the payoff matrices ($A_{G}$ and $A_{R}$), the interpolation factor *a*, the timescale parameter ϵ, and the carrying capacity *K* are identifiable, while δ and $\alpha_{G}$ remain unidentifiable; all the state variables are observable if *V* is the measured output. The CYTO model (or, more precisely, its first-order version, CYTO2) is fully identifiable and observable under the assumed measurements. The identifiability of the CICV depends on the measured variable: with the output *C*, all the parameters are identifiable; however, when *I* is the measured output, parameters like $r_{2}$, $\alpha_{1}$, $k_{1}$, $\alpha_{2}$, and $k_{2}$ become unidentifiable. It can be seen that the chemotherapy models show structurally better identifiability properties compared to the immunotherapy models.

Overall, the comparative analysis of Table 3, Table 4, Table 5 and Table 6 reveals clear differences among the model families. The classical growth models without treatments (Table 3) are fully identifiable and observable under natural measurement assumptions. In contrast, the radiotherapy models (Table 4) display more heterogeneity: while some formulations remain identifiable, the models RAD1 and RAD2 are not. The immunotherapy models (Table 5) show some structural limitations since many parameters are unidentifiable and several states are unobservable (especially CRS and IMRAD), which might be due to their increased complexity. Finally, the chemotherapy models (Table 6) tend to present favourable structural features, with most parameters and states identifiable from feasibly measurable outputs. These results suggest that models of increasing biological realism, especially in immunotherapy, are also those that pose more challenges for their identification.

From a practical point of view, unidentifiability means that there are infinite parameter sets that can reproduce the same measured outputs. As a result, it is impossible to determine the correct parameter estimates from experimental data with any degree of certainty; the results of a parameter estimation procedure are very likely to be wrong. In turn, due to the strong relation between (un)identifiability and (un)observability, it may also be impossible to infer the value of certain state variables, and their predictions may be unreliable. This limits the use of the model for therapy optimisation since treatment simulations could vary depending on the assumed parameter values.

## 5. Conclusions and Future Directions

In this paper, we have assembled a set of 20 dynamic models of tumour growth found in the literature. We have focused on mechanistic models that describe the physical and biochemical details of the processes involved in tumour growth using ODEs. Naturally, it would be unrealistic to attempt to include every model ever presented; instead, we have selected a number of them that include a wide array of mechanisms. Other models not included in this report can be analysed using the approach described here. Our selection includes some ODE models without therapeutic interventions, and others that describe the effect of treatments including radiotherapy, immunotherapy, and chemotherapy.

Our comparative results highlight clear differences between families of models: while classical growth laws and most chemotherapy models show favourable structural properties in regard to identifiability and observability, several immunotherapy models suffer from severe unidentifiability, at least for certain experimental configurations. These findings emphasise the importance of assessing structural properties before model calibration.

Overall, the abundance of models and the variety of types naturally lead to the question of how to choose the most appropriate one for a particular purpose. The decision should take into account, among other factors, the level of detail required and the availability of the data that is necessary to constrain the model. To facilitate this choice, for each model, we have discussed the measurements that are typically available, or feasible, and how they relate to the model variables. Sometimes, there may be more than one possible model that is compatible with the requirements; in this case, one may use a model selection technique to determine the one that provides the best balance between complexity and ability to fit the data [27].

Another important aspect to consider is whether the model allows us to estimate its unknown components reliably. This depends on two properties: identifiability, which refers to the ability to determine the model parameters from the available data, and observability, which refers to the ability to infer the internal variables from the measurements. If a model is not identifiable or observable, its predictions may be uncertain or misleading. Therefore, these properties should be analysed before applying a model, especially when it is used for designing treatments or making patient-specific predictions. In this paper, we have contributed to this endeavour by analysing the structural identifiability and observability (SIO) of the models. Since the SIO properties depend on the output functions, i.e., on the variables that are available for measurement, we have considered those possible combinations of measurements that are experimentally feasible. For some models, more than one output configuration is possible; in those cases, we have performed several analyses for each model. Our results, summarised in Table 3, Table 4, Table 5 and Table 6, shed light on our theoretical ability to obtain mechanistic insights from such models and can be used as a guide for choosing an appropriate model for a particular application.

The use of mechanistic models in oncology has been increasingly common since the 1990s. In recent years, these models have been instrumental in the development of personalised medicine. It is foreseeable that their use will only become more widespread in the near future as the key component of two emerging concepts: virtual patients and digital twins [52]. A virtual patient is basically a mathematical model calibrated with data obtained from a patient cohort. Thanks to this alignment with measurements, the simulations of a properly built virtual patient are expected to yield physiologically plausible outcomes. Hence, they can be used for simulating clinical trials for the purpose of drug development or therapy optimisation. The concept of digital twins [53] resembles that of virtual patients but with two key differences. First, a digital twin is typically built with data obtained from a single patient and is therefore a model of an individual, not of a target population. Second, digital twins are regularly fed with new data, thus enabling their use for cancer monitoring on a patient-specific basis. Digital twins are thus an evolution of virtual patient cohorts that are increasingly becoming an important part of precision medicine, enabling personalised predictions and in silico testing of therapeutic strategies [54]. For example, in a classic paper, Swiernak et al. [55] described virtual patient cohorts calibrated with clinical data to optimise chemotherapy protocols in breast cancer, demonstrating their potential to simulate clinical trials in silico. More recently, Ref. [56] discussed oncology digital twins that integrate longitudinal patient data to provide individualised treatment guidance and continuous monitoring.

Whereas mechanistic models are grounded in biological and physical understanding, recent research points to their strategic integration with data-driven approaches as a promising direction for the future. Unlike mechanistic models, data-driven models are not based on first principles and cannot be used for understanding a phenomenon, but they are capable of making predictions; however, the results lack explainability or interpretability. The emerging concept of mechanistic learning [57], which encompasses various ways of combining mechanistic models with machine and deep learning, offers a path to overcome some of the limitations that are inherent to each approach separately. Such hybrid methods promise to combine the interpretability of mechanistic models with the flexibility and data assimilation capabilities of learning-based algorithms. The incorporation of artificial intelligence (AI) techniques and big data to mechanistic models was recently discussed in [15]. Data-driven techniques can assist in estimating model parameters from high-dimensional data, correct residual errors, or serve as surrogates for complex models, thereby reducing computational costs and enabling real-time applications. The limitations of AI include the need for large datasets for training, which can typically be obtained only for patient cohorts and hence may fail to capture individual patient features. Crucially, AI predictions are difficult to generalise outside the conditions in which the training data was generated (overfitting to the training set). On the other hand, AI also offers important strengths for oncology modelling. Machine learning methods can detect complex patterns in high-dimensional clinical or omics data [58] and complement, as mentioned, mechanistic models in many different ways, such as predicting treatment responses [59]. Combined with mechanistic frameworks, AI can reduce computational costs by acting as surrogate models, enable real-time updating of digital twins, and facilitate the integration of heterogeneous data sources.

Moving forward, the field will benefit from a systematic exploration of different integration strategies, each suited to specific modelling goals, data availability, and clinical contexts. As data complexity and computational costs continue to grow, the combination of mechanistic and data-driven modelling has great potential to improve the predictive accuracy, adaptability, and relevance of tumour growth models.

## Figures and Tables

**Table 2:** Main features of the tumour growth models analysed in this paper.

Acronym	Parameters	States	Outputs	Inputs	Equations
EXP1	λ	*V*	*V*	–	(5)
ine EXP2	λ,K	*V*	*V*	–	(6)
ine EXP3	λ,K,θ	*V*	*V*	–	(7)
ine POW	a,γ	*N*	*N*	–	(8)
ine L–V	$\lambda_{S},\lambda_{R},K_{S},K_{R},\gamma_{R},\gamma_{S}$	$V_{S},V_{R}$	$V_{S},V_{R}$	–	(9) and (10)
ine GOM1	$a,b,N_{0}$	*N*	*N*	–	(11)
ine GOM2	α,K	*N*	*N*	–	(12)
ine LOG	a,K	*N*	*N*	–	(13)
ine BERT	a,b,γ	*N*	*N*	–	(14)
ine RAD1	$\lambda ,\alpha ,\beta ,d,t_{i}$	*V*	*V*	*d*	(15)
ine RAD2	$\lambda ,K,\theta ,\alpha ,\beta ,d,t_{i}$	*V*	*V*	*d*	(16)
ine RCAP	$\lambda ,K,\gamma ,t_{i}$	*V*	*V*	$t_{i}$	(17)
ine NECR	$\lambda ,K,\eta ,\gamma ,\zeta ,t_{i}$	$V_{t},N_{t}$	$V_{t},N_{t}$	$t_{i}$	(18) and (19)
ine CRS	$\eta ,\mu_{I},\nu ,\kappa ,\epsilon ,\theta ,\mu_{E},\mu_{P}$	$C_{I},C_{E},C_{P},$			(20)–(27)
	$\rho ,\gamma ,g_{0},K,\sigma_{M},\beta_{B},\beta_{K}$	$T_{P},T_{N},M_{a},$	$IL_{6}$	–	
	$\beta_{C},\delta_{M},\sigma_{I},\alpha ,\delta_{I},A,B,C$	$M_{i},IL_{6}$			
ine HCART	ϕ,ρ,θ,α,ϵ,μ,r,b,γ	$C_{T},C_{M},T$	*T*	–	(28)–(30)
ine IMRAD	$\lambda_{1},\lambda_{2},K_{C},K_{T},p,p_{1},\omega ,$			$K_{C}$, $\omega_{i}$	(31)–(37)
	$\phi ,a,\hat{T},\hat{T_{a}},\hat{T_{b}},\rho ,\psi ,s,$	$C\left(t\right),C_{d}\left(t\right)$	$C,\hat{A}$	$K_{T}$, $\delta_{c4}$	
	$q,\iota ,\eta ,\tau_{1},\tau_{2},\sigma ,b,c_{4}$	$T_{a}\left(t\right),\hat{A}\left(t\right)$		$\delta_{p1}$, $i_{c4},i_{p1}$	
	$\nu ,\mu ,i_{c_{4}},i_{p_{1}},h,t_{c_{4}},t_{p_{1}}$	$c_{4}\left(t\right),p_{1}\left(t\right),\hat{T}\left(t\right)$			
ine LEUK	$p_{T},k_{T},p_{NA},p_{AN}$	$T,T_{N},E$	T,E	e(t)	(38)–(45)
	$e\left(t\right),f,h,p_{E},d_{E},g$				
ine CYCLE	ε, δ, *K*, *a*, C(t), θ(t), $A_{G}$, $A_{R}$	$x_{1}$, $x_{2}$, *n*, *V*	$x_{2},V$	C(t), θ(t)	(46)–(56)
ine CYTO	$\phi ,\dot{\phi },\ddot{\phi },a,b$	*V*	V	–	(57)
ine CYTO2	$\phi ,\dot{\phi },\ddot{\phi },a,b$	$x_{1},x_{2}$	$x_{2}$	$\phi ,\dot{\phi },\ddot{\phi }$	(58) and (59)
ine CICV	$r_{1},r_{2},\alpha_{1},\alpha_{2},\alpha_{3},k_{1}k_{2}\beta_{1},\beta_{2},\gamma$	C,I	*C*	$\beta_{1},\gamma$	(60) and (61)

**Table 3:** Identifiability and observability summary for the tumour growth models without treatment.

Acronym	Known Inputs	Identifiable Parameters	Unidentifiable Parameters	Observable States	Unobservable States	Measured Outputs
EXP	-	All	-	All	-	*V*
ine POW	-	All	-	All	-	*N*
ine L–V	-	$\lambda_{S},\lambda_{R},K_{S}$	$K_{R},\gamma_{R},\gamma_{S}$	-	$V_{R}$	$V_{S}$
ine L–V	-	$\lambda_{S},\lambda_{R},K_{R}$	$K_{S},\gamma_{R},\gamma_{S}$	-	$V_{S}$	$V_{R}$
ine L–V	-	All	-	All	-	$V_{S}+V_{R}$
ine GOM	-	All	-	All	-	*N*
ine LOG	-	All	-	All	-	*N*
ine BERT	-	All	-	All	-	*N*

**Table 4:** Identifiability and observability summary for the tumour growth models with radiotherapy.

Acronym	Known Inputs	Identifiable Parameters	Unidentifiable Parameters	Observable States	Unobservable States	Measured Outputs
RAD1	δ	λ	α,β,d	All	-	*V*
ine RAD2	δ	λ,K,θ	α	All	-	*V*
ine RCAP	δ	All	–	All	–	*V*
ine NECR	δ	All	–	All	–	$V_{t}$
ine NECR	δ	All	–	All	–	$N_{t}$

**Table 5:** Identifiability and observability summary for the tumour growth models with immunotherapy.

Acronym	Known Inputs	Identifiable Parameters	Unidentifiable Parameters	Observable States	Unobservable States	Measured Outputs
CRS	–	η, $\mu_{I}$, κ, ϵ θ, $\mu_{E}$, $\mu_{P}$, ρ γ, $\delta_{M}$, $\sigma_{I}$, $\delta_{I}$ $g_{0}$	ν, *K*, *A*, *B* *C*, $\sigma_{M}$, α, $\beta_{B}$ $\beta_{K}$, $\beta_{C}$	–	All	$IL_{6}$
HCART	–	ϵ, θ, α, μ, *r*, *b*	ϕ, ρ, γ	–	All	*T*
IMRAD	$K_{C}$, $\omega_{i}$ $K_{T}$, $\delta_{c4}$ $\delta_{p1}$, $i_{c4}$ $i_{p1}$	$\lambda_{1}$, $\lambda_{2}$, *p*, ϕ *a*, *b*, ρ, ψ σ, η, μ, ν *h*, *s*, *q*, ι	τ1, τ2	–	All	*C*, *A*
LEUK	–	$p_{NA}$, $p_{AN}$, $p_{E}$	$p_{T}$, $k_{T}$, *e*, *f* *h*, $d_{E}$, *g*	*T*, $T_{N}$, *E*	–	*T*, $T_{N}$, *E*

**Table 6:** Identifiability and observability summary for the tumour growth models with chemotherapy.

Acronym	Known Inputs	Identifiable Parameters	Unidentifiable Parameters	Observable States	Unobservable States	Measured Outputs
CYCLE	$C_{t}$, $\theta_{t}$	AG,AR,				
		a,ϵ,K	$\delta ,\alpha_{G}$	All	–	*V*
ine CYTO2	$\dot{\phi },\ddot{\phi },b$	All	–	All	–	*V*
ine CICV	$\beta_{1},\gamma$	All	–	All	–	C
ine CICV	$\beta_{1},\gamma$	$r_{1},\delta ,$ $\alpha_{3}\beta_{2}$	$r_{2},\alpha_{1}$ $k1,\alpha_{2},k_{2}$	–	*C*	*I*

## Data Availability

All files are provided in the models folder of the STRIKE-GOLDD toolbox, which was used to perform the analyses. The toolbox is open source and can be freely accessed from Github (https://github.com/afvillaverde/strike-goldd, accessed on 1 August 2025).

## Abbreviations

The following abbreviations are used in this manuscript:

ODE	Ordinary Differential Equation
CAR	Chimeric Antigen Receptor
CRS	Cytokine Release Syndrome
TME	Tumour Microenvironment
DAMP	Damage-Associated Molecular Pattern
CML	Chronic Myeloid Leukemia
AI	Artificial Intelligence
uPA	Urokinase-Type Plasminogen Activator
PAI-1	Plasminogen Activator Inhibitor-1
VN	Vitronectin
